# Clinical characteristics of invasive candidiasis in infants born before 30 weeks of gestation: a nested case series from a multicenter cohort study in the Netherlands and Belgium

**DOI:** 10.1007/s00431-025-06694-5

**Published:** 2026-01-10

**Authors:** Rimke R. de Kroon, Aranka J. van Wesemael, Mirjam M. van Weissenbruch, Tim de Meij, Hendrik J. Niemarkt

**Affiliations:** 1 Amsterdam Reproduction and Development Research Institute, Amsterdam UMC, Amsterdam, the Netherlands; 2 Amsterdam Gastroenterology Endocrinology Metabolism Research Institute, Amsterdam UMC, Amsterdam, the Netherlands; 3 Department of Pediatric Gastroenterology, Emma Children’s Hospital, Amsterdam UMC, Amsterdam, the Netherlands; 4 Neonatal Intensive Care Unit, Emma Children’s Hospital, Amsterdam UMC, Amsterdam, the Netherlands; 5 Neonatal Intensive Care Unit, Maxima Medical Centre, Veldhoven, the Netherlands; 6 Department of Electrical Engineering, TU Eindhoven, Eindhoven, the Netherlands

**Keywords:** Preterm infants, Bloodstream infection, Sepsis, Candida, Invasive candidiasis, Neonatal intensive care unit

## Abstract

Invasive candidiasis poses a serious risk to preterm infants, due to its rapidly progressive and severe clinical course, resulting in considerable mortality and long-term morbidity. Therefore, the aim of the current study was to assess the clinical characteristics of invasive candidiasis in very preterm infants to increase awareness among clinicians. A multicenter cohort study database was screened for infants, born in one of ten Neonatal Intensive Care Units (NICUs) in the Netherlands and Belgium between October 2014 and May 2025, with blood- and/or cerebrospinal fluid culture-proven invasive candidiasis (gestational age < 30 weeks) in the first 29 days of life. Clinical data were retrospectively collected. Out of 2.824 infants, 24 were diagnosed with invasive candidiasis (0.8%), most frequently caused by *Candida albicans* (83%). Affected infants demonstrated distinct clinical features: extreme prematurity (mean 25.7 weeks ± 9 days), low birth weight (mean 827 ± 198 g), vaginal delivery (88%), and sepsis and/or gastrointestinal disease prior to clinical onset (46%). In 58%, initiation of antifungal treatment was delayed. The disease course was generally severe with end-organ disseminated candidiasis (33%), need for invasive ventilation (58%), cardiorespiratory support (42%), and red blood cell and/or platelet transfusion (71% and 33%). Both C-reactive protein and platelet count at diagnosis were associated with fatal outcome (*p* = 0.040 and *p* = 0.010, respectively).

*Conclusion*: In infants with distinct clinical features, clinicians should maintain a high index of suspicion, particularly in NICUs with a higher incidence of *Candida* colonization and/or infection. Our findings underline the need for a rapid diagnostic test to reduce treatment delays and improve clinical outcomes.

**Table:** 

**What is known:** • *Invasive candidiasis poses a risk to preterm infants due to its rapidly progressive disease course, high mortality, and long-term morbidity.* • *A critical need exists to enhance vigilance among clinicians, enabling timely diagnosis, and initiation of targeted treatment.*
**What is new:** • *Affected infants are characterized by shared clinical features and substantial disease burden.* • *Frequent vaginal delivery in affected infants suggests that colonization and infection may result from vertical transmission, offering opportunities for early prevention.*

**Supplementary Information:**

The online version contains supplementary material available at 10.1007/s00431-025-06694-5.

## Introduction

Invasive candidiasis, defined as a *Candida* infection of the blood and/or cerebrospinal fluid (CSF), is a threat to preterm infants, particularly to those with a gestational age (GA) below 32 weeks [1]. Although the incidence is low (~ 4–8% of all sepsis episodes) [2], the clinical course is severe and rapidly progressive, with potential for *Candida* dissemination to various organs [1]. Invasive candidiasis is associated with mortality rates of up to 60% with long-term effects, including neurodevelopmental impairment, in survivors [1].

Preterm infants are particularly susceptible to develop invasive candidiasis due to their immature immune system and impaired barrier function [3]. *Candida* colonization can occur as early as birth [4]. The use of medical devices (e.g. central venous catheters and invasive ventilation) compromises skin and mucosal barriers, enabling translocation of *Candida* from colonized sites into the bloodstream [5]. Additionally, disruption of the gut microbiota by broad-spectrum antibiotics may promote intestinal overgrowth of *Candida* [6], potentially increasing the risk for disease through translocation.

Targeted treatment is often delayed, as empirical therapy for suspected sepsis typically does not include antifungal agents. The turn-around time for fungal cultures is long [7], which may delay treatment initiation [8]. Some clinicians opt for prophylactic antifungal treatment. However, its use varies, due to the generally high number needed-to-treat, depending on the baseline incidence, and potential emergence of resistant strains [9, 10]. While *Candida albicans* is most frequently isolated, there appears to be a global shift toward non-*albicans Candida* species, including the emerging *Candida auris*, further complicating routine prophylaxis and treatment [11].

The aim of the current study was to comprehensively assess clinical characteristics of invasive candidiasis in infants born before 30 weeks of gestation to increase awareness among clinicians.

## Methods

This case series is embedded in a prospective multicenter cohort study, for which daily fecal samples are collected in the first month of life (GA < 30 weeks from October 2014 until March 2021, < 28 weeks from March 2021 onwards) in 10 Neonatal Intensive Care Units (NICUs) in the Netherlands and Belgium. Exclusion criteria are chromosomal abnormalities and/or congenital gastrointestinal (GI) diseases. The study was approved by the local medical ethical review boards (protocol 2014.386, amendment A2016.363) and written informed consent was obtained from parents or legal guardians. The study database between October 2014 and May 2025 was screened for infants with invasive candidiasis, defined as a positive blood and/or CSF culture for *Candida* resulting in systemic infection. For all screened infants, key baseline characteristics (GA, BW, mode of delivery) and sepsis and/or GI disease were obtained (Figure S1), facilitating direct comparison between affected infants and the rest of the cohort. For all affected infants, detailed clinical characteristics were retrospectively collected. Statistical analyses are described in Appendix A. Routine prophylaxis with fluconazole was administered in 1/10 study sites (Table S1). Treatment delay was defined as antifungal treatment initiation ≥ 1 days after withdrawal of blood culture for suspected infection. End-organ disseminated disease was defined as invasive candidiasis spread to distant organs. Culture-proven sepsis was defined as a blood and/or CSF culture-proven infection within or after the first 72 h of life (early-onset (EOS) and late-onset sepsis (LOS), respectively). GI disease included necrotizing enterocolitis (NEC) modified Bell’s stage ≥  2 A and/or focal intestinal perforation (FIP).

## Results

Out of 2.824 screened infants, 24 (0.8%) were diagnosed with invasive candidiasis (Figure S1, Table S2) with affected infants evenly distributed over the study period. Baseline characteristics of the affected infants are displayed in Table 1. Notably, the mean GA was 25 weeks + 5 days (SD ± 9 days), BW was 827 g (SD ± 198 g) and 89% was born through vaginal delivery. The three key baseline parameters were significantly different in the affected infants compared to the rest of the cohort (Table S3). The effects of adhering to two or more key baseline parameters (GA < 27 weeks, BW < 1000 g, and vaginal delivery) on the likelihood to develop invasive candidiasis was assessed, and demonstrated an increased risk (OR = 6.57, CI = 2.09–20.66,* p* < 0.001) (Table S4). Invasive candidiasis incidence decreased with increasing GA, from 3.7% at 24–25 weeks to 0.4% at 28–29 weeks.

**Table 1:** General patient characteristics and clinical characteristics of very preterm infants (gestational age < 30 weeks) with invasive candidiasis

	Infants diagnosed with invasive candidiasis (*n* = 24)
**General patient characteristics**	
**Demographics**	
Gestational age, mean (weeks + days) ± SD (days)	25 + 5 ± 9
Birth weight, mean (grams) ± SD (grams)	827 ± 198
Mode of delivery, vaginal delivery, n [%]	21 [88]
Biological sex, female, n [%]	9 [38]
Singleton, yes, n [%]	16 [66]
Apgar score at 5 min < 7, n [%]	8 [33]
**Medication practices**	
Received surfactant within 72 h after birth, n [%]	14 [58]
Ratio cumulative IV antibiotics in first month of life, days, median (IQR)	0.74 (0.33)
Exposure to IV antibiotics prior to onset of disease, n [%]	23 [96]
Exposure to probiotics prior to onset of disease, n [%]	7 [29]
**Feeding practices**	
Reached FEF in first month of life^1^, n [%]	16 [66]
Time to FEF, days, median (IQR)	13 (11)
Received exclusively human milk at first day of FEF^2^, n [%]	24 [100]
**Mortality**	
29-day mortality, n [%]	6 [25]
Mortality during NICU admittance, n [%]	10 [42]
**CLINICAL CHARACTERISTICS**	
**Diagnosis**	
Postnatal age at disease onset (days)^3^, median (IQR)	11 (14)
Onset of invasive candidiasis	
- Early onset (< 72 h), n [%]	2 [8]
- Late onset (≥ 72 h), n [%]	22 [92]
Exposure to invasive medical device (e.g. central venous or arterial catheters, invasive ventilation, umbilical lines, and/or peripheral IVs) in 48 h prior to disease onset, n[%]	23 [96]
Exposure to solely a peripheral IV prior to onset of disease, n [%]	5 [20]
*Candida* colonization prior to disease onset^4^:	
- Fungal pathogen cultured from urine, n [%]	3 [13]
- Fungal skin infection that required treatment with miconazole, n [%]	9 [38]
- Enteral nystatine administration, n [%]	2 [9]
Previous comorbidities:	
- Clinical sepsis, n [%]	3 [13]
- Bacterial culture-proven sepsis, n [%]	6 [25]
- Gastrointestinal disease (e.g. NEC or FIP), n [%]	6 [25]
- Absence of comorbidities, n [%]	13 [54]
**Microbiology**	
*Candida* cultured from:	
- Only blood culture, n [%]	20 [83]
- Only CSF culture, n [%]	2 [8.3]
- Both blood and CSF cultures, n [%]	2 [8.3]
Cultured pathogen:	
*- Candida albicans*, n [%]	20 [83]
*- Candida tropicalis*, n [%]	1 [4.2]
*- Candida parapsilosis*, n [%]	1 [4.2]
*- Candida glabrata*, n [%]	1 [4.2]
*- Candida guillermondii*, n [%]	1 [4.2]
Resistant strain isolated^5^, n [%]	2 [10]
Co-infection with bacterial pathogens^6^, n [%]	3 [17]
**Antifungal Treatment**	
Received fluconazole IV prior to disease onset^7^:	
- Routine prophylactic fluconazole, n [%]	2 [8]
- Clinical indication for fluconazole before diagnostic work-up, n [%]	5 [21]
- No exposure to fluconazole iv prior to diagnostic work-up, n [%]	17 [71]
Delay in initiation of IV antifungal treatment^8^, n[%]	14 [58]
Time to start IV antifungal treatment (days), in case treatment had not yet been initiated prior to diagnostic workup:	
- On the same day, n [%]	3 [13]
- 1 day after work-up, n [%]	6 [25]
- 2 days after work-up, n [%]	4 [16]
- 3 days after work-up, n [%]	3 [13]
- Mortality prior to start antifungal treatment, n [%]^9^	1 [4.2]
Antifungal treatment:	
- Monotherapy, n [%]	14 [58]
- Dual therapy, n [%]	6 [25]
- Triple therapy, n [%]	2 [8.3]
- Received no treatment due to mortality, n [%]	1 [4.2]
Type of IV antifungal treatment^10^:	
- Fluconazole, n [%]	22 [96]
- Liposomal amphotericin B, n [%]	7 [30]
- Micafungin, n [%]	2 [8.7]
- Caspofungin, n [%]	1 [4.3]
**Disease severity**	
Laboratory findings^11^:	
- C-reactive protein, mg/L, median (IQR; min, max)	28 (68; 0, 221)
- Platelets, 10^9^/L, median (IQR; min, max)	85 (177; 21, 560)
- Leukocytes, 10^9^/L, median (IQR; min, max)	24 (17; 5, 55)
Requirement for invasive ventilation, n [%]	14 [58]
Requirement for hemodynamic support:	
- Inotropics (e.g. hydrocortisone, dopamine, dobutamine), n [%]	8 [36]
- IV fluid resuscitation, n [%]	10 [42]
- Inotropics and/or IV resuscitation, n [%]	10 [42]
Requirement for transfusion:	
- Red blood cell transfusion, n [%]	17 [71]
- Fresh frozen plasma transfusion, n [%]	4 [17]
- Platelet transfusion, n [%]	8 [33]
End-organ disseminated candidiasis^12^:	
- Liver, n [%]	2 [8.3]
- Kidneys, n [%]	2 [8.3]
- Eyes, n [%]	1 [4.2]
- Joints, n [%]	1 [4.2]
- Central nervous system, n [%]	6 [25]
Absence of concurrent comorbidities, n [%]	16 [66]

^1^8 infants did not reach FEF (mortality prior to FEF (*n* = 6), reached FEF after first month of life (*n* = 2), respectively). ^2^Exclusively human milk may consist solely of mother’s own milk, donor milk, or a combination. ^3^The day on which the diagnostic work-up was performed, marked by at least a positive blood and/or CSF culture for *Candida*, was considered the day of onset. ^4^Missing data: nystatine administration (*n* = 1), urine cultures (*n* = 1). ^5^*C. guillermondii* was isolated with resistance to anidulafungin and micafungin (*n* = 1). *C. albicans* was isolated with resistance to fluconazole and voriconazole (*n* = 1). Missing data: 4 infants. ^6^Co-infection with *Escherichia coli *(*n* = 1), *Staphylococcus aureus* (*n* = 1), and Coagulase-negative *Staphylococci* (*n* = 2). ^7^Among the two infants who received fluconazole prophylaxis before developing invasive candidiasis, one had a blood culture positive for *C. albicans* resistant to both fluconazole and voriconazole, while the other had a blood culture positive for non-resistant *C. albicans.* ^8^Treatment delay was defined as initiation of antifungal medication on day after work-up or at a later timepoint. ^9^The infant (born with GA 26–27 weeks) who passed away without initiation of antifungal therapy died on the day the diagnostic work-up was conducted in the third week of life. ^10^Dosage per antifungal medication according to the local protocols: fluconazole 25 mg/kg/day on day 1 followed by 12 mg/kg/day from day 2, liposomal amphotericin B 3–5 mg/kg/day, micafungin 10 mg/kg/day, and caspofungin 2 mg/kg/day. ^11^For all three parameters, the most aberrant measurement within 48 h following work-up was collected. Missing data: CRP (*n* = 1), platelets (*n* = 1), leukocytes (*n* = 2). ^12^Dissemination to the liver, kidneys, and heart was confirmed by ultrasound. Ocular involvement was assessed through an ophthalmologic examination. Joint dissemination was established by a positive *Candida* culture of the synovial fluid. Central nervous system dissemination was confirmed by a positive culture of the CSF and/or by cerebral ultrasound findings. *Abbreviations:* *CRP* C-reactive protein, *CSF* cerebrospinal fluid, *FEF* full enteral feeding, *FIP* focal intestinal perforation, *IV* intravenous, *IQR* interquartile range, *NEC* necrotizing enterocolitis; *NICU* Neonatal Intensive Care Unit, *SD* standard deviation

### Diagnosis

Detailed clinical characteristics were obtained from all infants with invasive candidiasis (*n* = 24, Table 1). The median postnatal age was 11 days (IQR 14) at time of diagnostic work-up. *Candida* colonization was present in 46% prior to clinical onset. 46% was diagnosed with clinical or culture-proven sepsis (38%) and/or GI disease (25%) prior to clinical onset. The 29-day incidence of GI disease was higher in affected infants (46%) compared to those with non-*Candida* sepsis (16%) or no sepsis (9%, *p* < 0.001).

### Microbiology

*C. albicans* was the predominant isolated *Candida* species (83%) (Table 1). From 2 infants, a resistant strain was isolated (*Candida guillermondii* resistant to anidulafungin and micafungin and *C. albicans* resistant to fluconazole and voriconazole, respectively).

### Antifungal treatment

In 7 infants (29%), fluconazole had already been administered prior to empirical antibiotics, either as part of routine prophylaxis or based on clinical indicators, such as skin lesions or severe NEC (Table 1). Initiation of antifungal treatment was delayed in 58% of infants. In 3 infants (13%), initiation of adequate treatment took up to 3 days.

### Disease severity

Invasive ventilation and hemodynamic support was required in 58% and 42% of infants, respectively (Table 1). Platelets were low (median 85 × 10^9^/L, IQR 177) and 33% received a platelet transfusion. Moreover, 71% of infants received a red blood cell transfusion. C-reactive protein (CRP) was higher in the group with a fatal outcome (median 68 mg/L (IQR 112) in deceased infants vs. 7 mg/L (IQR 74) in survivors, *p* = 0.040), while platelets were lower (median 53 × 10^9^/L (IQR 55) in deceased infants vs. 124 × 10^9^/L (IQR 245) in survivors, *p* = 0.010). In 33%, disseminated candidiasis was diagnosed. Central nervous system involvement was most common (25%). Notably, 42% died during NICU admittance.

## Discussion

We described a case series of infants born before 30 weeks of gestation with invasive candidiasis. These infants generally had low GA, extremely low BW, and were born vaginally. Adherence to two or more key baseline criteria (GA < 27 weeks, BW < 1000 g, and vaginal delivery) was strongly associated with the likelihood of invasive candidiasis (OR = 6.57, CI = 2.09–20.66, *p* < 0.001), indicating that these features contribute to disease vulnerability. Moreover, affected infants were frequently exposed to clinical and/or culture-proven sepsis (38%) and/or GI disease (25%) prior to onset, and the 29-day GI disease incidence was significantly higher in the affected infants. Extremely preterm infants have a more immature immune system and impaired barrier function [3], which, combined with previous comorbidities, potentially facilitates disease development. Vaginal delivery may result in *Candida* colonization, particularly in the presence of maternal urogenital colonization, allowing vertical transmission [12]. The observed interval between birth and onset of disease (median 11 days postnatal age) could reflect the time required for birth-acquired colonization to progress to invasive disease. However, the potential role of vertical transmission in invasive candidiasis should be interpreted cautiously as, for example, maternal colonization status was not assessed in this study.

*Candida* as a cause for sepsis is often considered late in the diagnostic evaluation. However, timely consideration is of importance, considering that early initiation of (empirical) antifungal treatment is essential to ensure favorable outcomes [8]. Our study highlights that treatment delay occurred in 58%, which may in part be explained by the long turnaround time for fungal blood cultures [7], as well as the guidelines on standard empirical antimicrobial therapy for suspected sepsis in the Netherlands and Belgium that solely includes antibacterial agents. Antifungal treatment is frequently deferred until invasive candidiasis is confirmed by blood and/or CSF-culture results, resulting in delayed initiation of therapy for invasive candidiasis relative to the onset of clinical symptoms.

Given the rarity of invasive candidiasis, routine screening or empirical treatment with antifungals in all preterm infants is not justified. Our findings suggest that in infants with distinct clinical features (extreme prematurity, extremely low BW, vaginal delivery, and sepsis and/or GI disease prior to clinical onset), clinicians should maintain a high index of suspicion, particularly in units with a higher incidence of *Candida* colonization and/or infection. These findings may support clinicians in considering antifungal therapy, when two or more baseline criteria are met, as part of the empirical treatment regimen for infants with suspected sepsis. In countries with a different pathogen distribution and/or resistance patterns, alternative antifungal treatment (e.g. micafungin, amphotericin B) should be considered as empirical therapy, as opposed to fluconazole, while awaiting culture results. Notably, some infants developed invasive candidiasis despite receiving antifungal prophylaxis, emphasizing that prophylaxis alone should not reassure clinicians. When invasive candidiasis is suspected or confirmed, dissemination assessment, including CSF analysis and imaging of liver, brain, kidneys, eyes, and musculoskeletal system, should be considered as one third of cases had disseminated disease. This advice is supported by the severe disease progression, frequently requiring invasive ventilation, hemodynamic support, and red blood cell and/or platelet transfusion [8].

Our study stands out due its multicenter approach and detailed clinical characterization of invasive candidiasis. We were able to assess key baseline characteristics as well as sepsis and/or GI disease in the total cohort. However, additional clinical data from the unaffected infants was unavailable, hampering the identification of additional risk factors. Nonetheless, we observed relevant trends warranting further investigation and identified parameters (CRP and platelets) associated with a fatal outcome. Notably, future (prospective) studies should include additional maternal data collection, including fungal colonization and pregnancy complications, such as chorioamnionitis. Extrapolation of our findings to other countries may be challenging, one of the reasons being that *C. albicans* was the most common pathogen, hampering direct comparison with countries where non-*albicans* species are a larger contributor [11]. Lastly, our study focused on blood- and CSF-culture proven invasive candidiasis, and therefore may underestimate the overall burden of *Candida*-related disease by excluding other clinically significant manifestations of candidiasis, such as urinary tract infections, peritonitis, and congenital cutaneous candiasis.

In conclusion, this study provides a detailed clinical profile of infants with invasive candidiasis, highlighting shared clinical characteristics, delayed antifungal treatment, and substantial disease burden. These findings may increase awareness among clinicians and support the development of strategies for earlier recognition and targeted diagnostics. Our observation that invasive candidiasis more frequently occurs following vaginal delivery suggests that colonization and subsequent invasive infection may result from vertical transmission. This finding potentially opens new avenues for understanding early pathogenesis and potentially tailoring preventive strategies.

## Supplementary Information

Below is the link to the electronic supplementary material.

Supplementary file1 (DOCX 88.8 KB)

## Data Availability

The data that support the findings of this study are available on request from the corresponding author. The data are not publicly available due to privacy restrictions.

## Declarations

This study was conducted in accordance with the ethical principles of the Declaration of Helsinki. The study was approved by the Medisch Ethische Toetsingscommissie (METC), the Dutch equivalent of the Institutional Review Broad, of the VU medical center (protocol 2014.386, amendment A2016.363). Written informed consent was obtained from parent(s) or legal guardian(s) for all infants.

## Abbreviations

*β*: Estimate coefficient
*BW*: Birth weight
*CI*: Confidence interval
*CSF*: Cerebrospinal fluid
*CRP*: C-reactive protein
*EOS*: Early-onset sepsis
*FIP*: Focal intestinal perforation
*GA*: Gestational age
*GI*: Gastrointestinal
*IQR*: Interquartile range
*LOS*: Late-onset sepsis
*NEC*: Necrotizing enterocolitis
*NICU*: Neonatal intensive care unit
*OR*: Odds ratio
*SD*: Standard deviation
*SE*: Standard error
*Stand resid*: Standardized residual
