# Supplementary material for: Clinical characteristics of invasive candidiasis in infants born before 30 weeks of gestation: a nested case series from a multicenter cohort study in the Netherlands and Belgium
Source: Eur J Pediatr. 2026 Jan 10;185(2):70. doi: 10.1007/s00431-025-06694-5 (PMC12790506; doi:10.1007/s00431-025-06694-5)
Supplement: Supplementary file 1 — Supplementary file1 (DOCX 88.8 KB) [file 431_2025_6694_MOESM1_ESM.docx]

**Supplementary materials**

Corresponding manuscript title: *Clinical characteristics of invasive candidiasis in infants born before 30 weeks of gestation: a nested case series from a multicenter cohort study in the Netherlands and Belgium*

Authors:
Rimke R. de Kroon, Aranka J. van Wesemael, on behalf of the generation P studygroup, Mirjam M. van Weissenbruch, Tim de Meij, Hendrik J. Niemarkt

**Supplementary Figure 1: Overview of inclusion process & clinical data collection**

The figure displays the inclusion process and clinical data collection for the current study. Out of 2.824 screened preterm infants (gestational age <30 weeks), born between October 2014 and May 2025, 24 infants (0.8%) were diagnosed with blood and/or cerebrospinal fluid culture-proven invasive candidiasis in the first month of life. For the total cohort, both affected and unaffected infants, general clinical data was collected, including key baseline parameters (gestational age, birth weight, and mode of delivery) as well as comorbidities in the first month of life (both culture-proven sepsis as well as gastrointestinal disease, defined as necrotizing enterocolitis stage 2A or higher and/or focal intestinal perforation). For the infants diagnosed with invasive candidiasis, detailed clinical data collection was conducted, including general patient characteristics as well as a thorough description of the disease episode, including information on diagnosis, microbiology, antifungal treatment, and disease severity. *Created in BioRender. Amsterdamumc, Eminds. (2025) https://BioRender.com/ayfqndz*

**
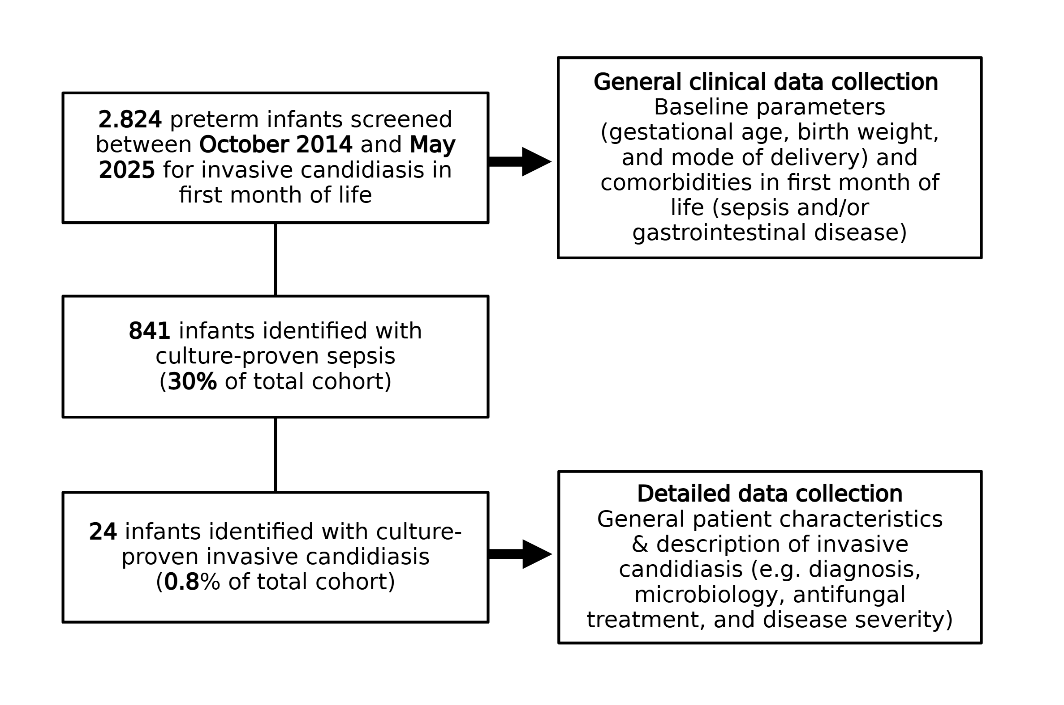
**

**Supplemental Table 1: Routine antifungal prophylaxis strategies across study site participating in the current study.**

This table summarizes the administration of fluconazole prophylaxis across study sites as defined by local protocols, providing context for-site variability in antifungal prophylaxis strategies. Table displays that only 1 out of 10 study sites administrated routine prophylaxis based on gestational age.

| **Study site** | **Prophylaxis^1^** | **Indication described in local study site protocol** |
| --- | --- | --- |
| 1 | No | Not applicable |
| 2 | No | Not applicable |
| 3 | No | Not applicable |
| 4 | Fluconazole, iv | In case of multiple concurrent risk factors^2^ |
| 5 | Fluconazole, iv or nystatine, oral | In case of multiple concurrent risk factors^2^ |
| 6 | No | Not applicable |
| 7 | Nystatine, oral | In case of fecal *Candida* colonization |
| 8 | No | Not applicable |
| 9 | No | Not applicable |
| 10 | Yes | Standard prophylaxis for all infants with GA <26 weeks |
| ^1^For fluconazole (iv), the standard dosage used in all study sites for prophylaxis is 6 mg/kg/72h in 1 dosage (PNA <14 days) and 6 mg/kg/48h in 1 dosage (PNA 14-28 days); for nystatine (oral), the standard dosage used in all study sites is 100.000 IE/day in 1 dosage. ^2^The specific risk factors taken into account to determine eligibility for antifungal prophylaxis differ per study site. *Abbreviations: BW = birth weight; GA = gestational age.* | | |

**Supplemental Table 2: Total incidence and site-specific incidence of invasive candidiasis in infants born before 30 weeks of gestation.**

This table the total number of screened infants, infants with blood- and/or CSF culture-proven invasive candidiasis, and incidence of invasive candidiasis per study site and in total. The table demonstrates that the overall incidence combining the 10 study sites is 0.8%, ranging from 0% to 1.7% in the individual study sites.

| **Study site** | **Total screened infants** | **Infants with invasive candidiasis** | **Incidence (%)** |
| --- | --- | --- | --- |
| 1 | 534 | 9 | 1.7 |
| 2 | 293 | 2 | 0.7 |
| 3 | 461 | 0 | 0.0 |
| 4 | 146 | 2 | 1.4 |
| 5 | 197 | 1 | 0.5 |
| 6 | 177 | 1 | 0.6 |
| 7 | 278 | 1 | 0.4 |
| 8 | 291 | 5 | 1.7 |
| 9 | 306 | 2 | 0.7 |
| 10 | 141 | 1 | 0.7 |
| Total | 2824 | 24 | 0.8 |

**Supplemental Table 3: Comparison of three key baseline parameters of infants with invasive candidiasis, non-*Candida* sepsis, and without sepsis.**

|  | **Invasive candidiasis (n=24)** | **Non-*Candida* sepsis (n=811)** | **No sepsis (n=1969)** | ***p*-value^1^** | **Post-hoc^2^** |
| --- | --- | --- | --- | --- | --- |
| **Gestational Age** | | | | | |
| Gestational age, mean (weeks+days)±SD (days) | 25+5±9 | 26+5±11 | 27+3±11 | <0.001* | 1 vs 3: <0.001* 2 vs 3: <0.001* 2 vs 1: <0.003* |
| **Birth Weight** | | | | | |
| Birth weight, mean (days)±SD (days) | 827±198 | 916±247 | 1011±262 | <0.001* | 1 vs 3: 0.002* 2 vs 3: <0.001* 2 vs 1: 0.221 |
| **Mode of Delivery** | | | | | |
| Mode of delivery, vaginal delivery, n [%] | 21 (88) | 371 (46) | 884 (44) | <0.001* | 1: std resid 4.15, sign 2: std resid 0.16, n.s. 3: std resid -1.00, n.s. |
| ^1^For normally distributed continuous variables, ANOVA was conducted; for categorical variables, Chi square was conducted. *p*-value ≤0.05 was considered significant. For 20 infants in the total cohort (n=2.824 infants, <1% missing data), one or more of the three key baseline parameters were not available and these infants were therefore excluded from this analysis. ^2^The three subgroups were defined as following in the post-hoc analysis: infants with invasive candidiasis (1), non-*Candida* sepsis (2), and no sepsis (3). For continuous parameters, Tukey’s Honest Significant Difference was conducted; for categorical parameters, standard residuals were calculated. *p*-value ≤0.05 was considered significant. For the latter, residuals were depicted for the following categories per subgroup: <27 weeks for gestational age (GA), <1000 grams for birth weight (BW), and vaginal delivery for mode of delivery. *Abbreviations:* *BW = birth weight; GA = gestational age; std residual = standardized residual.* | | | | | |

**Supplemental Table 4: Logistic regression to assess the effects of adhering to two or more key baseline parameters on the likelihood that preterm infants develop invasive candidiasis within the first month of life.**

| **Predictor^1^** | **β** | **SE** | **Z value** | **OR** | **95% CI for OR** | ***p*-value** |
| --- | --- | --- | --- | --- | --- | --- |
| Intercept | -5.978 | 0.501 | -11.94 | n.a. | n.a. | <0.001* |
| Adhering to 2 or more key baseline criteria | 1.883 | 0.549 | 3.43 | 6.57 | 2.09-20.66 | <0.001* |
| ^1^A logistic regression was performed to assess the effects of adhering to two or more key baseline criteria (gestational age <27 weeks, birth weight <1000 grams, and born through vaginal delivery) on the likelihood that infants develop invasive candidiasis in the first month of life. Results demonstrate that the logistic regression model was statistically significant,  χ^2^(1) = 16.34, p<0.001 (Likelihood ratio test). The model explained 0.059% (Nagelkerke R^2^) of the variance in the outcome. *Abbreviations: CI = confidence interval; OR = Odds Ratio; SE = standard error.* | | | | | | |

**Appendix A**

Statistical analysis

All statistical analyses were performed using R (version 4.4.3) or SPPS (version 28). Continuous variables are presented as mean ± standard deviation (SD) or median (interquartile range, IQR) depending on the distribution.

Categorical variables are presented as counts and percentages. For comparison of continuous parameters between two groups, Mann-Whitney U or student t-test was used, depending on the distribution. For comparison of continuous variables across two or more groups, one-way ANOVA was used or Kruskal-Wallis H test, depending on the distribution. Tukey’s Honest Significance Difference test was used for post-hoc pairwise comparisons. Categorical variables were compared using **Pearson’s chi-squared test. Post-hoc pairwise comparisons** were conducted for categorical variables by examining the **standardized residuals (stand resid).** Residuals > |1.96| were considered statistically significant at *p*≤0.05.

A univariable logistic regression was conducted to assess the association between adhering to two or more key baseline criteria (defined as GA <27 weeks, BW <1000 grams, and born through vaginal delivery) and the likelihood that infants develop invasive candidiasis in the first month of life. Model coefficients (β), standard errors (SE), z values, odds ratios (OR), and 95% confidence intervals (CI) were reported. The model fit was evaluated using the likelihood ratio test comparing the full model to the intercept-only model. R² was calculated using Nagelkerke’s method. Statistical significance was defined as *p*≤0.05.
